# Impact of Genetic Reduction of NMNAT2 on Chemotherapy-Induced Losses in Cell Viability *In Vitro* and Peripheral Neuropathy *In Vivo*

**DOI:** 10.1371/journal.pone.0147620

**Published:** 2016-01-25

**Authors:** Richard A. Slivicki, Yousuf O. Ali, Hui-Chen Lu, Andrea G. Hohmann

**Affiliations:** 1 Program in Neuroscience, Indiana University, Bloomington, IN, United States of America; 2 Department of Psychological and Brain Sciences, Indiana University, Bloomington, IN, United States of America; 3 The Cain Foundation Laboratories, Jan and Dan Duncan Neurological Research Institute at Texas Children’s Hospital, Houston, TX 77030, United States of America; 4 Department of Pediatrics, Baylor College of Medicine, Houston, TX 77030, United States of America; University of Sao Paulo, BRAZIL

## Abstract

Nicotinamide mononucleotide adenylyl transferases (NMNATs) are essential neuronal maintenance factors postulated to preserve neuronal function and protect against axonal degeneration in various neurodegenerative disease states. We used *in vitro* and *in vivo* approaches to assess the impact of NMNAT2 reduction on cellular and physiological functions induced by treatment with a vinca alkaloid (vincristine) and a taxane-based (paclitaxel) chemotherapeutic agent. NMNAT2 null (NMNAT2-/-) mutant mice die at birth and cannot be used to probe functions of NMNAT2 in adult animals. Nonetheless, primary cortical cultures derived from NMNAT2-/- embryos showed reduced cell viability in response to either vincristine or paclitaxel treatment whereas those derived from NMNAT2 heterozygous (NMNAT2+/-) mice were preferentially sensitive to vincristine-induced degeneration. Adult NMNAT2+/- mice, which survive to adulthood, exhibited a 50% reduction of NMNAT2 protein levels in dorsal root ganglia relative to wildtype (WT) mice with no change in levels of other NMNAT isoforms (NMNAT1 or NMNAT3), NMNAT enzyme activity (i.e. NAD/NADH levels) or microtubule associated protein-2 (MAP2) or neurofilament protein levels. We therefore compared the impact of NMNAT2 knockdown on the development and maintenance of chemotherapy-induced peripheral neuropathy induced by vincristine and paclitaxel treatment using NMNAT2+/- and WT mice. NMNAT2+/- did not differ from WT mice in either the development or maintenance of either mechanical or cold allodynia induced by either vincristine or paclitaxel treatment. Intradermal injection of capsaicin, the pungent ingredient in hot chili peppers, produced equivalent hypersensitivity in NMNAT2+/- and WT mice receiving vehicle in lieu of paclitaxel. Capsaicin-evoked hypersensitivity was enhanced by prior paclitaxel treatment but did not differ in either NMNAT2+/- or WT mice. Thus, capsaicin failed to unmask differences in nociceptive behaviors in either paclitaxel-treated or paclitaxel-untreated NMNAT2+/- and WT mice. Moreover, no differences in motor behavior were detected between genotypes in the rotarod test. Our studies do not preclude the possibility that complete knockout of NMNAT2 in a conditional knockout animal could unmask a role for NMNAT2 in protection against detrimental effects of chemotherapeutic treatment.

## Introduction

Nicotinamide mononucleotide adenylyl transferases (NMNATs) are neuronal maintenance factors postulated to preserve normal neuronal function and protect neurons from insult [1]. NMNATs are essential enzymes that condensate adenosine triphosphate (ATP) with either nicotinamide mononucleotide (NMN) or nicotinic acid mononucleotide (NaMN) to produce nicotinamide adenine dinucelotide (NAD) or nicotinic acid adenine dinucleotide (NaAD) [1]. Mammals have three different NMNAT genes. NMNAT1 is primarily localized to the nucleus, whereas NMNAT2 and NMNAT3 are localized to the golgi apparatus and mitochondria, respectively [2]. NMNATs maintain upkeep and repair of axons, and overexpression of these proteins may confer neuroprotection in specific disease states [3]. NNMNAT2 has been implicated as an essential factor for axonal survival in primary sensory and sympathetic nerve cell injury models *in vitro* [4,5]. Complete loss-of-function of NMNAT2 *in vivo* has been shown to be lethal, where mice die at birth, due to severe peripheral denervation. Hence, NMNAT2 plays an essential role in maintaining the integrity of peripheral neurons.

NMNAT2 is depleted in distal ends of injured axons before signs of Wallerian-like degeneration appear ([4,6]; reviewed in [1]). NMNAT2 depletion produces neurodegeneration in uninjured axons that is absent following knock-down of NMNAT1 or NMNAT3 [4]. Exogenous NMNAT2 expression thus offers axonal protection *in vitro* and both rescues and delays axon degeneration in a *Drosophila* nerve injury model [7]. Because NMNAT2 is detected in synaptosomes prepared from cortical neurons [8] it may play a role in the maintenance of synaptic function. Consequently, depletion of NMNAT2 could be implicated in peripheral neuropathies where synaptic loss is prevalent [8]. Given its indispensable role in axonal and neuronal maintenance, we hypothesized that NMNAT2 depletion may impact the severity of chemotherapy-induced peripheral neuropathies.

All major classes of chemotherapeutic agents produce dose limiting peripheral neuropathies [9]. Although vincristine and paclitaxel induce anti-tumor actions through distinct mechanisms [10–12] [13], both agents produce behavioral hypersensitivities (i.e. mechanical and cold allodynia) in rodents that mimic clinical symptoms of chemotherapy-induced peripheral neuropathy [14–16]. NMNAT2-/- mice die at birth and display impaired axonal growth in both peripheral and central neurons [6,17]. We, therefore, used NMNAT2+/- mice, which exhibit 50% reductions in NMNAT2 protein levels [17] and survive to adulthood, to investigate the possible contributions of NMNAT2 to both the development and maintenance of neuropathic pain induced by chemotherapeutic treatment. First, we used cell culture to compare the impact of complete (100%) and partial (50%) NMNAT2 depletion on neurotoxicity induced by vincristine and paclitaxel treatment using an MTT assay [18]. We examined the impact of NMNAT2 reduction on protein levels of known NMNAT isoforms (i.e. NMNAT1, NMNAT2 and NMNAT3) in dorsal root ganglia (DRG) derived from adult NMNAT2+/- and WT mice. We measured NAD/NADH levels and quantified microtubule associated protein 2 (MAP2) and neurofilament protein levels in adult DRG samples of adult NMNAT2+/- and WT mice to determine whether NMNAT2 reduction altered NMNAT enzyme activity or induced signs of neurodegeneration. Finally, we characterized the behavioral phenotype of NMNAT2+/- mice following challenge with different classes of chemotherapeutic agents. We compared the development and maintenance of chemotherapy-induced mechanical and cold allodynia induced by treatment with either vincristine or paclitaxel in NMNAT2+/- and WT mice. Possible differences in locomotor activity between genotypes were assessed using the rotarod test. Finally, NMNAT2+/- and WT mice treated with either paclitaxel or its vehicle were challenged with capsaicin, the pungent ingredient in hot chili peppers, in an attempt to unmask differences in nociceptive responding between genotypes.

## Methods

### Genotyping and Cortical Neuronal Cultures

**Fig 1:**
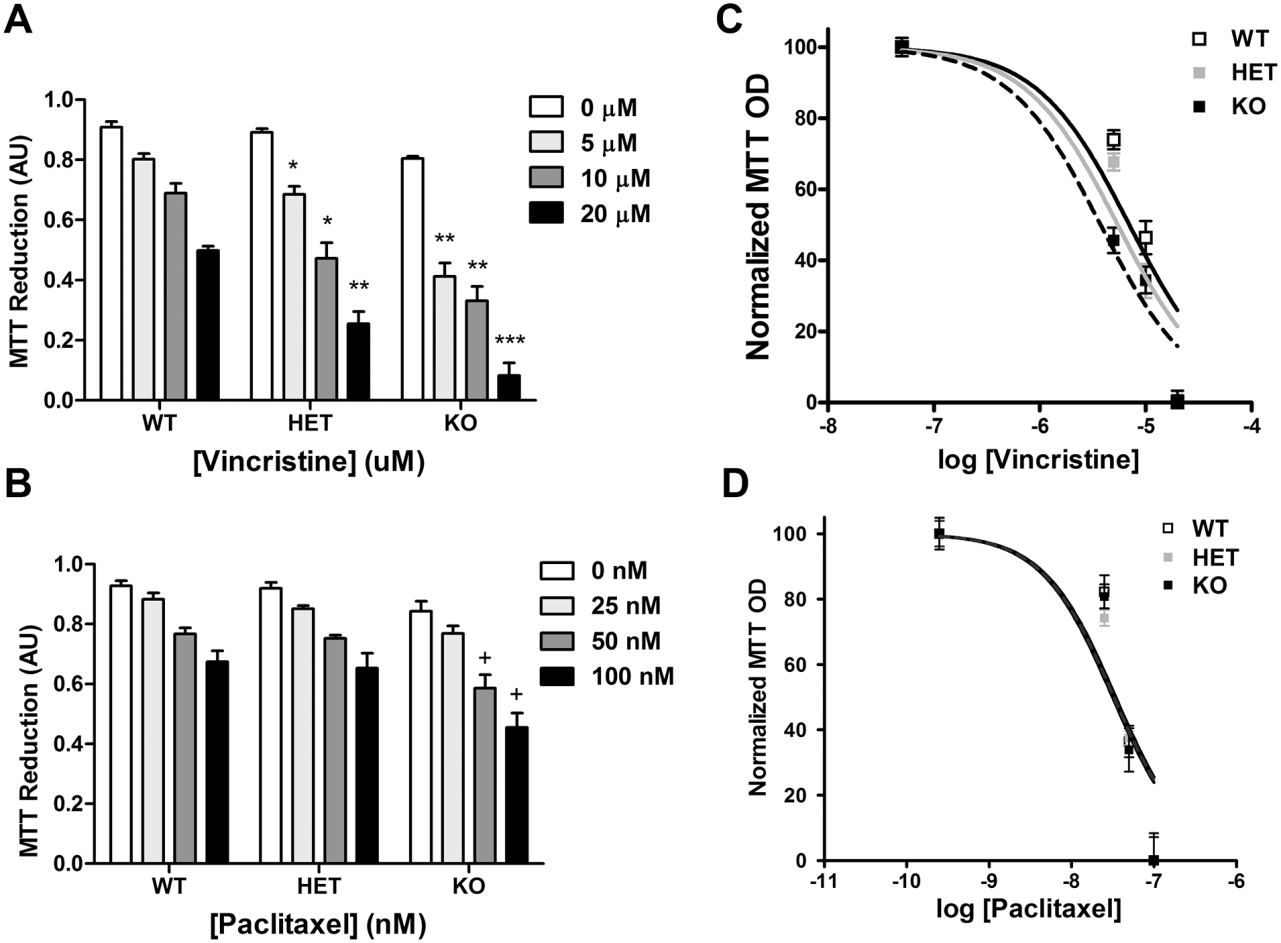
Treatment of NMNAT2-/- cortical neurons with vincristine and paclitaxel renders them hypersensitive to neurotoxicity. A) DIV14 cortical neurons (i.e. 14 days in vitro) from wildtype (WT), NMNAT2+/- (HET) and NMNAT2-/- (KO) mice were treated with increasing concentrations of vincristine for 12 hours. Vincristine dose-dependently reduced the viability of both NMNAT2+/- and NMNAT2 -/- neurons. NMNAT2-/- neurons were especially hypersensitive to vincristine-mediated toxicity. B) Treatment of these neurons with increasing concentrations of paclitaxel significantly reduced cell viability in NMNAT2-/- at higher concentrations only. Panels C) and D) represent normalized absorbance (OD) in MTT-treated primary neurons that were used to calculate EC_50_ values for chemotherapy-induced reductions in cell viability in WT, HET and KO conditions. (n = 3 in at least three independent experiments; *p<0.05, **p<0.001, ***p<0.0001 vs. each corresponding genotype; +p < 0.05 vs. WT).

Cortical neurons were prepared from the cortical plates of E16.5 embryos harvested from pregnant NMNAT2+/- dams. The pups were individually genotyped to determine if they were WT, NMNAT2+/- or NMNAT2-/- before harvesting the neurons from individual cortices as described [17]. Following genotyping, cortical neurons were harvested using the Worthington Papain Dissociation Kit (LK003153, Worthington Biochemical Corporation, Lakewood, NJ), according to the manufacturer’s protocol. Following proper dissociation of cortical neurons, cells from the embryos of same genotypes were merged and counted. 50,000 neurons were plated per well in Polylysine-D coated 96 well plates (BD Biosciences, San Jose, CA) in Neurobasal Media (Gibco, Grand Island, NY). Following plating, half of the media was replenished with B-27 and L-glutamine supplemented fresh Neurobasal media on Days-in-Vitro 2 (DIV2), DIV7 and DIV14 prior to treatment with vincristine or paclitaxel (see Fig 1 legend). Animal housing and use were in compliance with the NIH Guidelines for the Care and Use of Laboratory Animals and were approved by the institutional animal care committee at Baylor College of Medicine.

### MTT Assay

Neuronal sensitivity to chemotherapeutic agents was studied using the MTT assay, which measures cell viability, based upon the ability of viable cells to reduce the tetrazolium dye MTT 3-(4,5-dimethylthiazol-2-yl)-2,5-diphenyltetrazolium bromide to its insoluble form formazan. MTT assay was performed on DIV14 primary cortical neurons grown in 96 well plates, using Vybrant MTT Cell Proliferation Assay Kit, according to manufacturer’s guidelines (Life Technologies). After neuronal maturation, DIV14 neurons were treated with increasing doses of vincristine or paclitaxel for 12 hours and cell viability was measured following treatment. The absorbance values for cells treated with MTT were measured using the 540 nm wavelength, Opsys MR^™^ spectrophotometer, and Revelation Quicklink software. EC_50_s for vincristine and paclitaxel to suppress cell viability in WT, NMNAT+/- and NMNAT-/- mice were calculated from absorbance values measured in MTT-treated cells, normalized to those measured in absence of chemotherapeutic treatment.

### Adult DRG Isolation and Protein Extraction

Eight month old NMNAT2-+/- and WT littermates were euthanized by rapid decapitation to collect cervical DRGs (n = 4 mice per group). Dissected DRGs were minced into small pieces with a scissor and homogenized in RIPA buffer (50 mM Tris-HCl, pH 8.0, with 150 mM sodium chloride, 1.0% Igepal CA-630 (NP-40), 0.5% sodium deoxycholate, and 0.1% sodium dodecyl sulfate). The samples were homogenized with an electric pestle and further sonicated for clarity. Protein concentrations were calculated using Bradford Assay (BioRad).

### Western Blot Analysis

For analysis of protein expression levels, 20 μg of total protein lysate per sample were resolved by sodium dodecyl sulfate polyacrylamide gel electrophoresis and transferred onto a nitrocellulose membrane, and probed with antibodies against NMNAT1 (1:1000, SantraCruz Biotechnology, Dallas, TX), NMNAT2 (1:1000, Novus Biologicals, Littleton, CO), NMNAT3 (1:1000, Abcam, Cambridge, MA), Neurofilament (1:1000, Millipore, Temecula, CA), MAP2 (1/2000, Millipore, Temecula, CA) and GAPDH (1:5000, Millipore, Temecula, CA). Western blot analysis was performed with infrared dye conjugated secondary antibodies, IR700 and IR800 (1:10000, LI-COR Biosciences, Lincoln, NE). Blots were imaged and processed on an Odyssey^®^ Infrared Imaging System. Densitometry analysis was performed on all the blots using ImageJ software (NIH). Data are represented as means ± SEM.

### NAD/NADH Enzymatic Assay

To measure the raw levels of NAD and NADH, DRG neurons were extracted from 5-month old NMNAT2+/- and WT littermates. Immediately post-harvest, DRGs were chopped into smaller pieces and homogenized in lysis buffer provided by the Amplite Colorimetric Total NAD and NADH Assay Kit (AAT Bioquest, Sunnyvale, CA). Using the manufacturer’s protocol, raw NAD/NADH levels were measured using supplied standards and an absorbance microplate reader at ~576 nm.

### Subjects for Behavioral Studies

Fifty-four mice (32 male, 22 female) on the background strain OVE2171-P9kk4 weighing 21.6 to 67.7g at the beginning of testing were used. Subjects included thirty-three heterozygous (NMNAT2 +/-) and twenty-one WT mice. All animals were single housed in a temperature-controlled facility, with food and water *ad libitum*. Animals were maintained on a regular 12 h light/ 12 h dark cycle (lights from 7 am to 7pm). All procedures using adult mice were approved by Institutional Animal Care and Use Committee of Indiana University Bloomington and followed the guidelines for the treatment of animals of the International Association for the Study of Pain [19].

### Drugs and Chemicals

Paclitaxel was purchased from Tecoland Corporation (Edison, NJ, USA) and dissolved in a cremophor-based vehicle (1:1:18 ratio of cremophor EL/ethanol/saline). Vincristine sulfate was purchased from Tocris (Bristol, BS11 0QL, UK) and dissolved in saline. Capsaicin was purchased from Sigma-Aldrich (St. Louis, MO) and dissolved in a vehicle of 7% Tween 80 in 0.9% saline, sonicated, and filtered as described previously [20].

### General Behavioral Experimental Protocol

Animals were bred and genotyped at Baylor College of Medicine (Lu laboratory) and transferred to Indiana University for behavioral assessments. Following quarantine and habituation to the facility, *in vivo* testing was initiated (Hohmann laboratory). All experiments were conducted by an experimenter (RAS) blinded to the genotype and treatment condition.

In Experiment 1, mice were initially evaluated in the accelerating rotarod test to compare baseline motor coordination in NMNAT2+/- and WT mice.

In Experiment 2, twenty-eight mice were randomly assigned to receive either 14 once daily injections of vincristine sulfate (0.1 mg/kg i.p. per day x 14 days) or saline vehicle. Sensitivities to mechanical and cold stimulation were evaluated on days 0, 4, 8, 12, 16, 20 and 24 following initiation of vincristine or saline dosing.

In Experiment 3, twenty-six mice were randomly assigned to receive either paclitaxel (4 mg/kg i.p.) or cremophor-based vehicle as described in our previously published work [16]. Injections were performed four times on alternate days (i.e. day 0, 2, 4 and 8). Mechanical paw withdrawal thresholds and time spent attending to the acetone stimulated paw were assessed. In all studies, when injections and behavioral testing occurred on the same day, behavioral testing was always performed prior to injection of the chemotherapeutic agent or its vehicle.

In Experiment 4, both paclitaxel-treated and paclitaxel-untreated (i.e. cremophor vehicle-treated) mice were challenged with an intradermal injection (10 μl) of capsaicin (1 μg). Capsaicin was injected unilaterally into the plantar surface of the left hind paw 24 days post initiation of paclitaxel (or cremophor vehicle) dosing, when paclitaxel-induced allodynia was established and stable. Duration of nocifensive behavior (i.e. time spent licking, lifting and flinching the injected paw) was quantified over 5 minutes following intraplantar injection. The threshold for paw withdrawal to punctate mechanical stimulation was assessed with the same electro vonFrey anesthesiometer used to measure chemotherapy-induced mechanical allodynia. Responsiveness to mechanical stimulation was assessed in both paws before and 7, 60 and 120 minutes after capsaicin injection.

### Rotarod Test

An accelerating rotarod was employed to measure motor coordination in NMNAT2+/- and WT mice as described previously [20]. Mice were placed on a rotating drum 3 cm in diameter. The rotation increased from 4 to 40 rpm over a five-minute interval. The session stopped once the five-minute interval was reached, or the animal fell off the rotating drum. Training trials took place on two separate days prior to the test day, and consisted of three trials each. Mice that were unable to remain on the drum for at least 30 seconds did not meet the training criteria and were not included in the experiment. The amount of time the animals were able to remain on the drum was recorded.

### Assessment of Paw Withdrawal Thresholds to Mechanical Stimulation

Paw withdrawal thresholds to mechanical stimulation were measured using an electronic von Frey anesthesiometer (IITC model Alemo 2390–5, Woodland Hills, CA) as described in our previously published work [16]. Mice were placed on an elevated metal mesh table and were allowed to habituate under individual, inverted plastic cages to the testing platform for at least 20 minutes until exploratory behavior had ceased. After the habituation period, a force was applied to the midplantar region of the hind paw with a semiflexible tip connected to the anesthesiometer. Mechanical stimulation was terminated when the animal withdrew its paw and the value of the applied force was recorded in grams. Mechanical paw withdrawal thresholds were obtained in duplicate for each paw, and are reported as the mean of duplicate determinations obtained from each animal.

### Assessment of Cold Responsiveness

Hypersensitivity to cold stimulation was measured using the acetone method in triplicate in both paws using the same animals used to assess mechanical hypersensitivity; these methods are identical to those described in our previously published work [16]. Testing for responsiveness to acetone commenced 20 min after testing responsiveness to mechanical stimulation. Acetone was applied to the plantar surface of the hind paw through the end of a blunt one C.C. syringe hub. The total time the animal spent attending to the acetone-stimulated paw (i.e. elevation, shaking, or licking) was recorded over one minute following acetone application. Acetone was applied three times to each paw with a 3-minute interval between applications. Values for each animal were calculated as the mean of 6 determinations of acetone responsiveness derived from each mouse.

### Assessment of Nocifensive Behavior and Mechanical Paw Thresholds in Response to Capsaicin

Immediately after intraplantar (i.pl.) injection of capsaicin, nocifensive behavior (i.e. guarding, licking, lifting the injected paw) was measured (in sec) over 5 minutes. Mechanical paw withdrawal thresholds were recorded at 7, 60 and 120 min post injection. Mechanical paw withdrawal thresholds were measured in each paw in duplicate and averaged for paws ipsilateral and contralateral to capsaicin injection. Intraplantar injection of vehicle for capsaicin did not produce nocifensive behavior or mechanical hypersensitivity in mice over the same observation intervals in our previous studies (data not shown).

### Statistical Analyses

Paw withdrawal thresholds (mechanical) and duration of acetone-evoked behavior (cold) were calculated for each paw and averaged. Analysis of variance (ANOVA) for repeated measures was used to determine the time course of paclitaxel-induced mechanical and cold allodynia as well as capsaicin-evoked mechanical hypersensitivity. One-way ANOVA was used to identify the source of significant interactions at each time point, followed by Bonferroni *post hoc* tests (for comparisons between groups). Capsaicin-evoked nocifensive behavior was analyzed by a 2 x 2 ANOVA with genotype and chemotherapy condition as the factors. A priori comparisons were also made using planned comparison and paired t-tests, as appropriate. All statistical analyses were performed using IBM-SPSS Statistics version 19.0 (SPSS inc., an IBM company, Chicago, IL, USA). GraphPad Prism Software (San Diego, CA) was used to calculate EC_50_s and 95% confidence intervals in the in vitro studies. *P*< 0.05 was considered statistically significant.

## Results

### Loss of NMNAT2 in cortical neurons results in increased vulnerability to vincristine- and paclitaxel-mediated neurotoxicity

We used an MTT colorimetric assay to quantify the impact of vincristine and paclitaxel on cell viability in cultured cortical neurons derived from NMNAT2-/-, NMNAT2+/- and WT mice. Loss of NMNAT2 increased vulnerability of cortical neurons to increasing doses of vincristine, with the most robust effects observed in NMNAT2-/- neurons (Fig 1A and 1C). Vincristine-induced reductions in cell viability were observed in both WT (EC_50_ (95% confidence intervals): 7 μM (7.28, 9.74) and NMNAT2+/- (EC_50_: 5.46 μM (6.24, 7.94) neurons with NMNAT-/- neurons being almost non-viable at the highest vincristine concentrations (10 μM, p < 0.001; 50 μM, p < 0.0001) (EC_50_: 3.8μM (3.63, 6.63).

**Fig 2:**
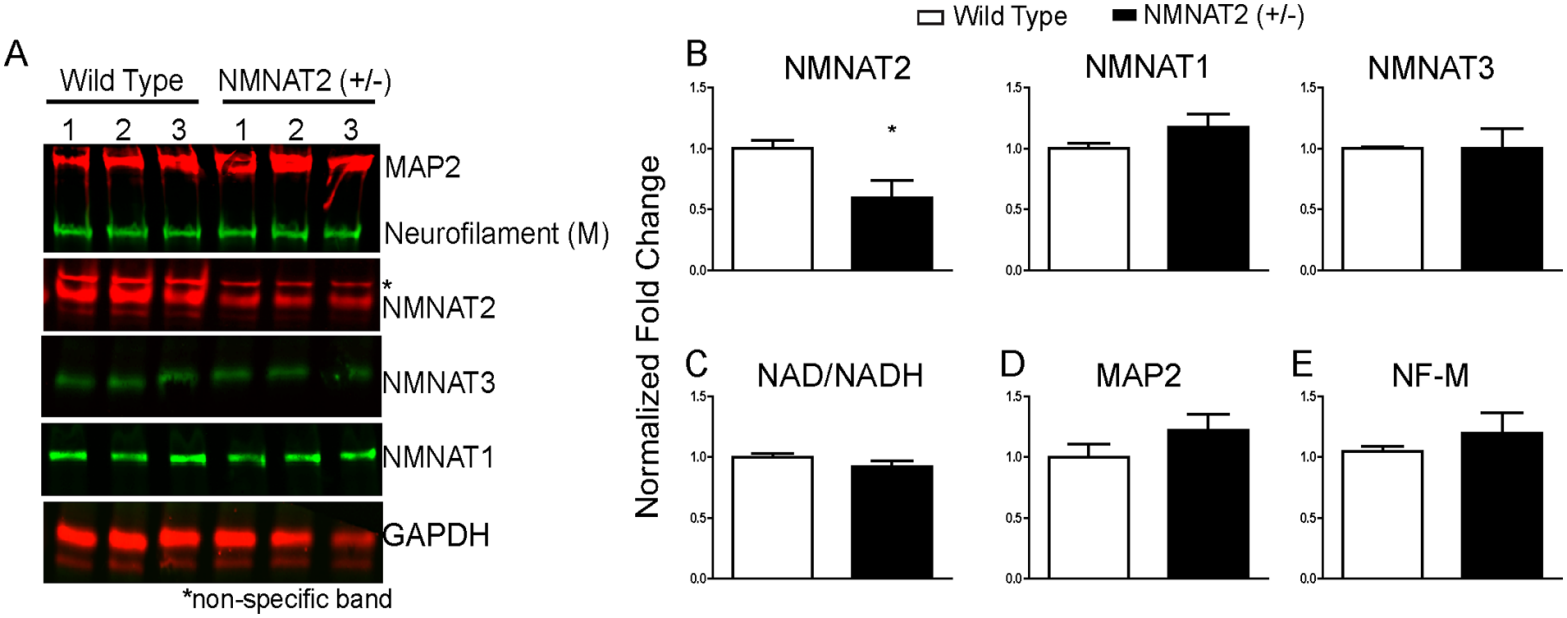
Dorsal root ganglia (DRG) derived from adult NMNAT2+/- mice do not display compensatory changes in other NMNAT levels, nor do they exhibit deficiencies in NAD/NADH activity or reductions in MAP2 or neurofilament levels compared to WT. A,B) DRG isolated from NMNAT2 +/- animals display a reduction in NMNAT2 levels but no changes in NMNAT1 or NMNAT3 levels. C) Average NAD/NADH activity levels do not differ in DRGs isolated from WT or NMNAT2+/- mice (shown as normalized NAD/NADH levels). D) microtubule associated protein 2 (MAP2) and neurofilament (NF-M) protein levels did not differ in NMNAT2+/- and WT mice (*p<0.05, **p<0.001, ***p<0.0001).

Paclitaxel decreased cell viability in NMNAT2-/- neurons at higher concentrations (50 nM, p < 0.05 and 100 nM, p < 0.05) (EC_50_ (95% confidence interval): 32.69 nM (34.61, 45.68)) and produced a non-significant trending decline in cell viability in both WT (EC_50_: 34.42 nM (35.79, 47.27) and NMNAT2+/- (EC_50_: 31.18 nM (32.76, 45.94) neurons (Fig 2B and 2D). Thus, complete loss of NMNAT2 function, which can be studied only *in vitro*, makes cortical neurons more vulnerable to the cytotoxic effects of effects of both vincristine and paclitaxel treatment.

### NMNAT2 haplo-insufficiency in adult DRGs do not impact NMNAT1 or 3 and does not cause spontaneous degeneration

DRGs derived from NMNAT2+/- mice exhibited a 50% decrease in endogenous NMNAT2 protein levels but no compensatory changes in the levels of NMNAT1 or NMNAT3 (Fig 2A and 2B). Moreover, normalized NAD/NADH activity levels, a measure of NMNAT enzyme function, did not differ in DRGs extracted from adult NMNAT2+/- and WT mice (Fig 2C). Normalized NAD levels (Mean ± S.E.M) were 0.955 ± 0.0513 vs. 1.0 ± 0.093 for NMNAT2+/- vs. WT, respectively whereas NADH levels (Mean ± S.E.M) were 0.893 ± 0.120 vs. 1.0 ± 0.039 for NMNAT2+/- vs. WT, respectively). Furthermore, neither MAP2 (Fig 2D) or neurofilament (Fig 2E) protein levels were altered in DRG derived from adult NMNAT2+/- and WT mice.

### NMNAT2 haplo-insufficiency does not alter baseline responding or produce motor ataxia

Body weight did not differ between genotypes either before or after chemotherapeutic treatments with either vincristine (p > 0.30) or paclitaxel (p > 0.99). No significant differences were observed between male and female mice on any dependent measure assessed within either genotype in any study; therefore data from both sexes were pooled into a single experimental group for each genotype.

**Fig 3:**
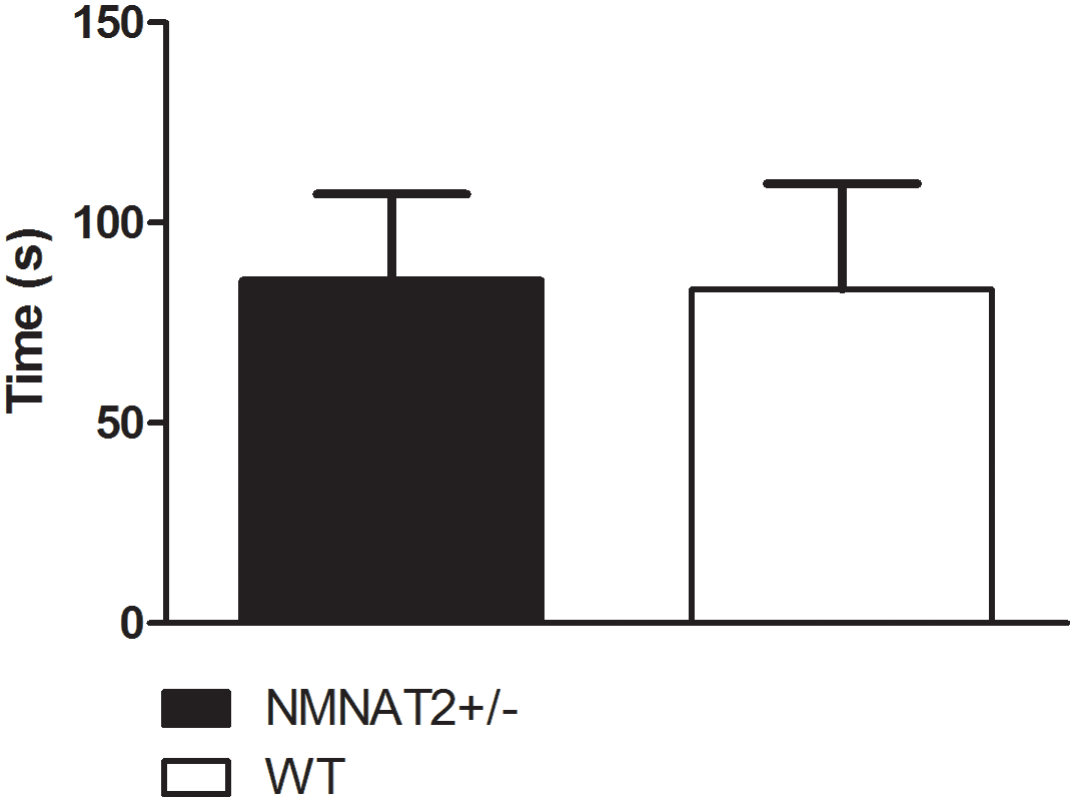
Latency to descend from the rotatrod did not differ between NMNAT2+/- and WT mice. Data are expressed as mean ± SEM (NMNAT2 +/- n = 16, WT n = 10). * p < .05 vs. vehicle, one-way analysis of variance followed by Bonferroni post hoc test.

Rotarod descent latencies did not differ between genotypes during any of the three rotarod training trials (F_2,23_ = .184, p > .83). An increase in time spent on the rotarod across time was observed, consistent with improvement in performance over training trials (F_2,48_ = 3.825, p < .03). However, this effect did not differ as a function of genotype. Moreover, NMNAT2 +/- and WT mice did not differ in the latency to descend from the rotating drum in the rotarod test on the test day (i.e. following completion of training trials) (p > .85; Fig 3). Prior to vincristine or paclitaxel treatment, paw withdrawal thresholds to mechanical stimulation or duration of acetone-evoked behaviors did not differ between genotypes (p > .05 for all experiments) in any study.

### The development and maintenance of vincristine-induced neuropathic pain do not differ in NMNAT2+/- and WT mice

**Fig 4:**
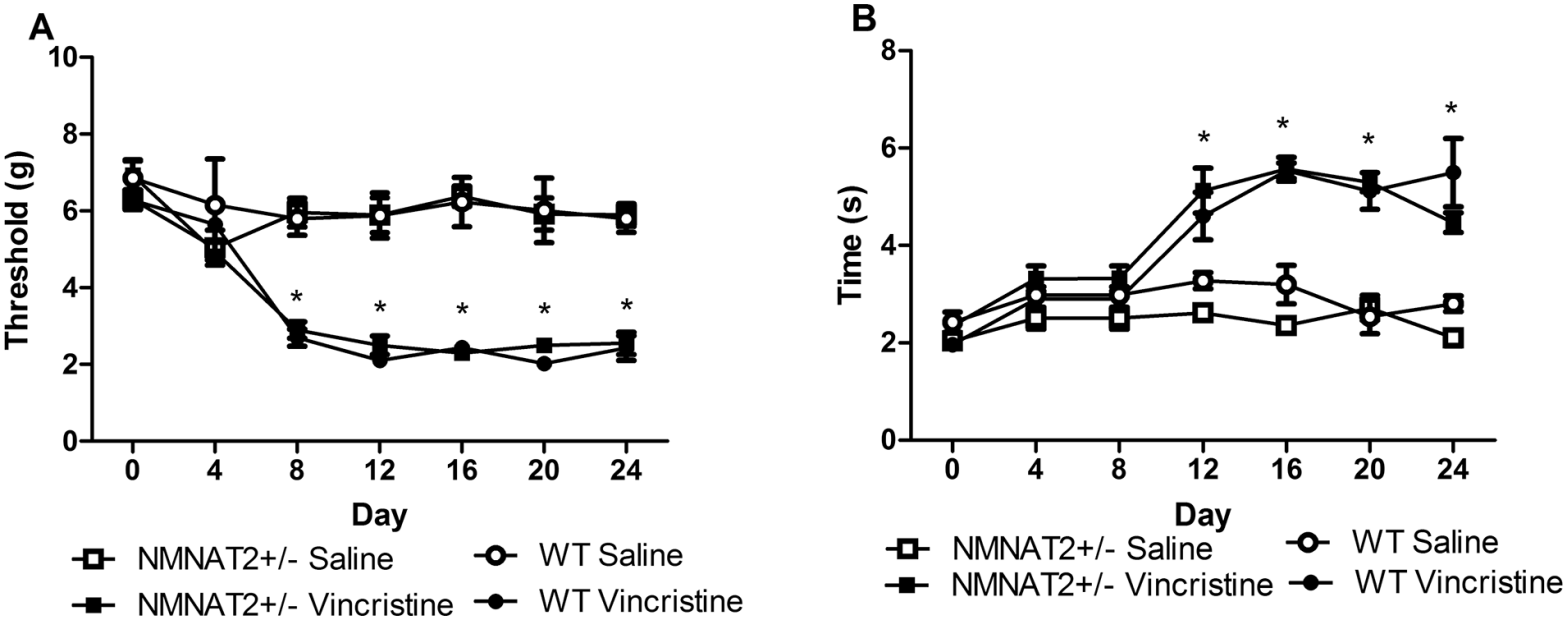
Vincristine-induced mechanical and cold allodynia did not differ between NMNAT2+/- and WT mice. There were no differences between genotypes in the development or maintenance of vincristine-induced mechanical hypersensitivity (A) or cold allodynia (B). Data are expressed as mean ± SEM (NMNAT2+/- vehicle n = 8, NMNAT 2+/- vincristine = 9, WT Vehicle = 5, WT vincristine = 6) * p < .05 vs. control, one-way analysis of variance followed by Bonferroni post hoc test.

Vincristine decreased paw withdrawal thresholds (F_3,24_ = 59.979, p < 0.001) and increased acetone-evoked behaviors (F_3,24_ = 51.821, p < 0.001), consistent with the development of mechanical and cold allodynia. No differences were observed between NMNAT2+/- and WT mice in the development or maintenance of vincristine-induced hypersensitivity to mechanical (p = 1.00; Fig 4A) or cold (p > .15; Fig 4B) stimulation. Moreover, no differences in mechanical and cold responsiveness were observed between NMNAT2+/- and WT mice that received saline in lieu of vincristine (Fig 4A and 4B).

### The development and maintenance of paclitaxel-induced neuropathic pain do not differ in NMNAT2+/- and WT mice

**Fig 5:**
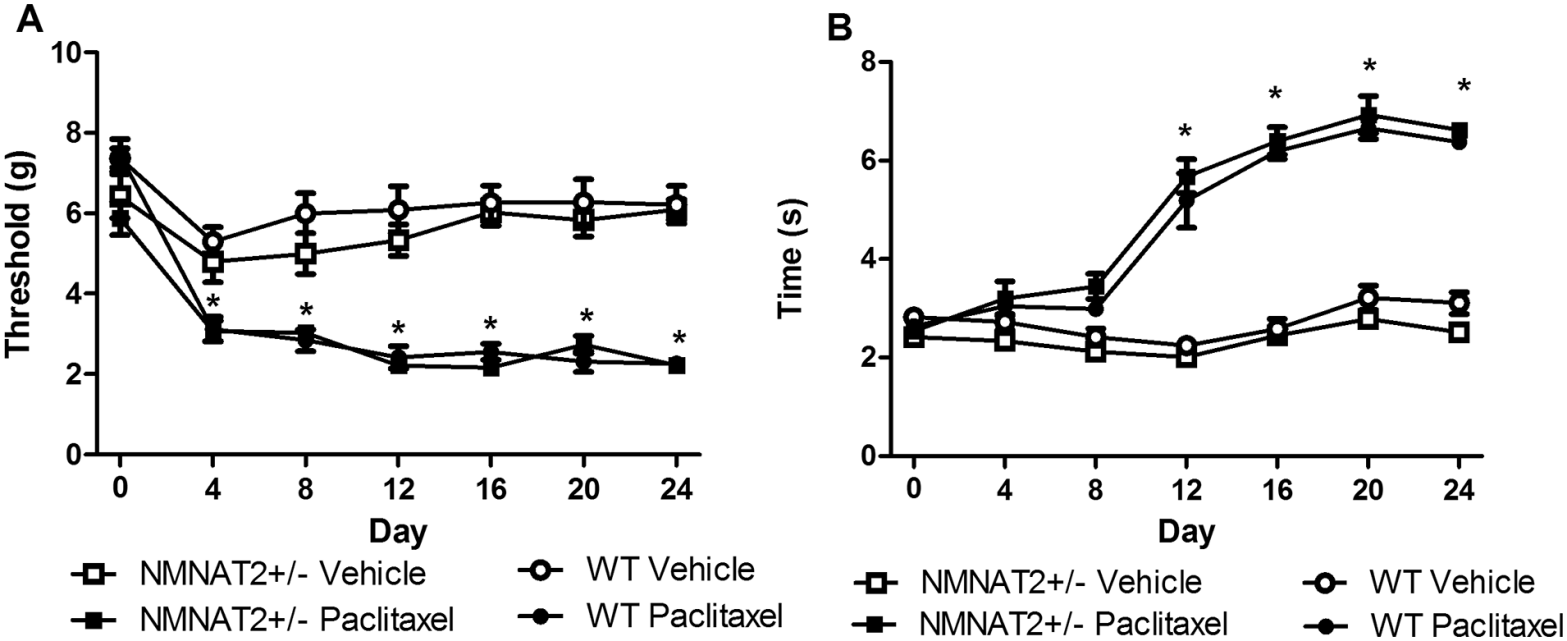
Paclitaxel-induced mechanical and cold allodynia did not differ between NMNAT2+/- and WT mice. There were no differences between genotypes in the development or maintenance of paclitaxel-induced mechanical hypersensitivity (A) or cold allodynia (B). (NMNAT2 +/-vehicle n = 8; NMNAT2+/- paclitaxel n = 8; WT vehicle n = 5; WT paclitaxel = 5) * p < .05 vs. control, one-way analysis of variance followed by Bonferroni post hoc test.

Paclitaxel decreased paw withdrawal thresholds (F_3,22_ = 48.141, p < 0.001) and increased acetone-evoked behaviors (F_3,22_ = 144.518, p < 0.001), consistent with the development of mechanical (Fig 5A) and cold (Fig 5B) allodynia. No differences were detected between NMNAT2+/- and WT mice treated similarly with either paclitaxel or its vehicle on any dependent measure assessed. NMNAT2 +/- and WT mice did not differ in either the development or maintenance of paclitaxel-induced hypersensitivity to mechanical (p > .55; Fig 5A) or cold stimulation (p > .68; Fig 5B).

### Capsaicin challenge does not unmask differences in nociceptive responding in NMNAT2+/- or WT mice with prior histories of either paclitaxel or cremophor vehicle treatment

**Fig 6:**
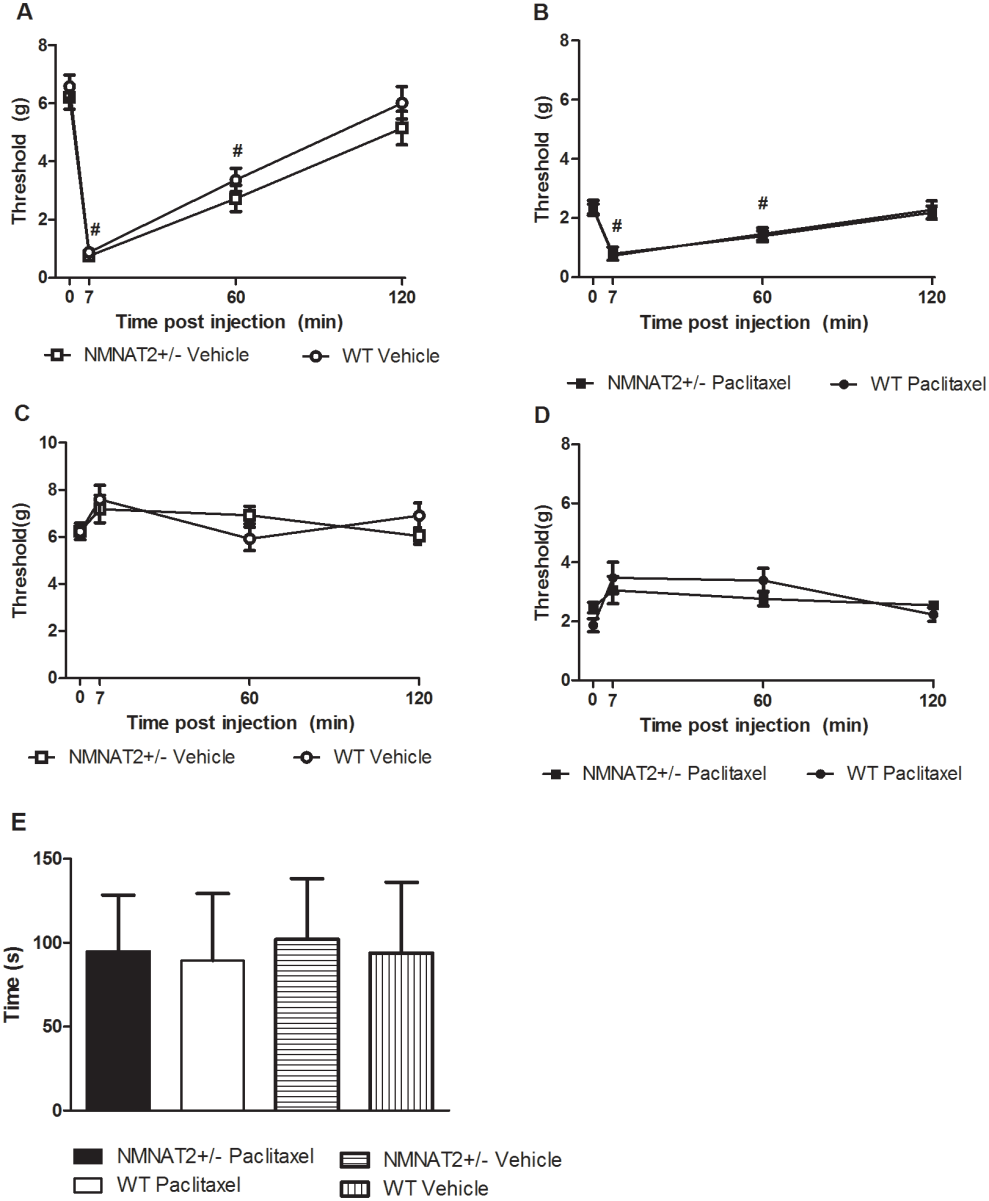
Capsaicin-evoked mechanical hypersensitivity and nocifensive behavior did not differ between NMNAT2+/- and WT mice with identical histories of either paclitaxel or cremophor vehicle treatment. No differences were observed between genotypes in mechanical paw withdrawal thresholds assessed ipsilateral (A, B) or contralateral (C, D) to capsaicin injection in either paclitaxel-treated (B, D) or paclitaxel-untreated (receiving cremophor-vehicle) (A, C) mice. Capsaicin-evoked nocifensive behavior did not differ between groups (E). Data are expressed as mean ± SEM (NMNAT2 +/- vehicle n = 8, NMNAT 2+/- paclitaxel n = 8, WT vehicle n = 5, WT paclitaxel = 5). # p < .05 vs. pre-capsaicin baseline, repeated measures ANOVA.

Paclitaxel treatment increased responsiveness to mechanical stimulation prior to challenge with intradermal capsaicin (F_3,22_ = 584.614, p < 0.001). Following capsaicin challenge, an increase in hypersensitivity to mechanical stimulation was observed in both vehicle-treated (p < .001; Fig 6A) and paclitaxel-treated mice (p < .002; Fig 6B); hypersensitivity was observed at 7 and 60 minutes following capsaicin injection but was no longer present at 120 minutes following capsaicin challenge (p > .061; Fig 6A and 6B).There were no differences between genotypes in mechanical hypersensitivity in either the capsaicin-injected (p = 1.00; Fig 6A and 6B) or non-injected paws (p > .42; Fig 6C and 6D). Similarly, capsaicin-evoked nocifensive behavior did not differ between NMNAT2+/- or WT mice in either paclitaxel- treated or paclitaxel-untreated (i.e.cremophor vehicle-treated mice) (F_3,22_ = 584.614, p = 1.00; Fig 6E).

## Discussion

We hypothesized that depletion of NMNAT2 would exacerbate both neurotoxicity and peripheral neuropathy induced by chemotherapeutic treatment. Our in vitro studies provide only partial support for this hypothesis whereas our *in vivo* studies fail to support a protective role for NMNAT2 in chemotherapy-induced peripheral neuropathy. To overcome the lethality-at-birth issue in NMNAT2-/- mice, we studied the effects of vinca and taxane-based chemotherapeutic agents in cortical neurons cultured from NMNAT2-/-, NMNAT2+/- and WT embryos. These embryonic NMNAT2-/- neurons can survive in culture and mature despite problems with stunted neurite outgrowth [21]. When challenged with vincristine, cortical neurons lacking NMNAT2 displayed a marked reduction in cell viability compared to NMNAT2+/- and WT neurons. This conclusion is also supported by the differences in EC_50_ values for vincristine to suppress primary cortical neuron viability in our MTT assay in WT, NMNAT2+/- and NMNAT2-/- mice. Effects of NMNAT2 depletion on cell viability were more subdued following paclitaxel than vincristine treatment, possibly due to differences in mechanism of action between these agents. Thus, EC_50_ values for paclitaxel to suppress primary cortical neuron viability did not differ markedly between genotypes. Our *in vitro* findings may suggest a threshold for NMNAT2 levels, below which neuronal maintenance is totally hampered. It is important to emphasize that cortical neurons were not used in the present study to model DRG function, but rather as a model system that would allow us to productively compare impact of NMNAT2 depletion on cell viability using NMNAT2-/- mice (which die at birth) in comparison to NMNAT2+/- and WT mice. Paclitaxel also induces cell death in DRG neurons at similar doses to those tested in the present experiments [22]. The present observations nonetheless warrant development of conditional NMNAT2-/- mice, to permit further *in vivo* characterizations of NMNAT2 depletion while bypassing the lethality issues which prevented us from characterizing NMNAT2-/- mice in adulthood.

Chemotherapeutic drugs have been shown to impair axonal transport *in vitro* and *in vivo* [23,24]. A potential mechanism by which these microtubule-targeting drugs cause peripheral neuropathy could be a reduced supply of NMNAT2 into distal axons [25]. In our *in vitro* studies, complete loss of NMNAT2 in cultured cortical neurons disabled neuronal maintenance mechanisms that buffer against external toxicities arising from chemotherapeutic agents. Effects were more marked with vincristine, which produces anti-tumor effects by altering cytoskeletal structure and disrupting microtubules [10–12], than with paclitaxel, which impedes the cell cycle and stabilizes microtubules. Although both agents ultimately produce apoptosis and induce peripheral neuropathy [26], the mechanisms underlying development and maintenance of chemotherapy-induced neuropathy may be distinct from those underlying the anti-tumor properties of these agents [27].

Our studies provide the first *in vivo* characterization of the impact of NMNAT2 knockdown on behavior. In our studies, NMNAT2+/- mice did not differ from WT animals in terms of development or maintenance of neuropathic allodynia induced by either vincristine or paclitaxel treatment. NMNAT2+/- and WT animals showed vincristine-induced behavioral hypersensitivities that were comparable to their WT littermates. Moreover, there were no genotype differences in locomotor activity or basal nociceptive thresholds prior to chemotherapeutic treatment that could otherwise impact the behavioral phenotype. Previous *in vitro* studies, which report neurite loss due to vincristine treatment, have suggested upregulation of NMNAT2 as a possible preventative measure against vincristine-induced axonal deficits [28]. In our studies, NMNAT2+/- mice exhibited a 50% reduction of NMNAT2 protein in adult dorsal root ganglia relative to WT mice with no compensatory changes in other NMNAT isoforms (i.e. NMNAT1 and NMNAT3) and no change in NMNAT enzyme activity (i.e. as measured by NAD/NADH levels). Thus, compensatory changes in other NMNAT isoforms or NMNAT enzyme activity are unlikely to account for our failure to observe alterations in chemotherapy-induced peripheral neuropathy in NMNAT2+/- mice. NMNAT2+/- and WT mice also exhibit comparable levels of MAP2 or neurofilament levels in adult DRG. These findings suggest that adult NMNAT2+/- mice do not exhibit a neurodegenerative phenotype characterized by reductions in DRG levels of MAP2 or neurofilament. More work is necessary to determine whether NMNAT2 levels or activity change over development in a manner that would differentially impact chemotherapy-induced changes in cell viability (measured in embryonic cultured cells) and chemotherapy-induced neuropathy (measured in adult mice).

NMNAT1 has been shown to prevent axon degeneration and peripheral neuropathy induced by both paclitaxel and vincristine, suggesting a Wallerian-like mechanism of axon degeneration [29]. NMNAT1 overexpression protects against vincristine-induced pathology in an *in vitro* model, albeit to a lesser extent than that observed with the Wallerian degeneration slow (Wld^s^) protein [29]. The Wld^s^ protein is more effective in delaying axonal damage in DRG cultures than NMNAT1 [28] and was reported to substitute for NMNAT2 in a NMNAT2-/- mice [6]. However, we do not observe compensatory changes in other NMNATs in DRG derived from adult NMNAT2+/- mice. Moreover, the failure to observe changes in neurofilament or MAP2 protein expression levels in the adult DRG is consistent with absence of spontaneous peripheral degeneration occurring in NMNAT2+/- DRG.

To our knowledge, this is the first study to examine the impact of NMNAT2 knockdown on the effects of paclitaxel either *in vivo* or *in vitro*. Paclitaxel is a chemotherapeutic agent commonly employed in the treatment of lung, breast and ovarian cancers [13]. Paclitaxel induces a reduction in neurite length and morphology changes in cultured neurons as well as axonal degeneration in biopsied patients [30]. However, similar to mice treated with vincristine, NMNAT2+/- mice did not exhibit any differences in paclitaxel-induced mechanical or cold allodynia relative to WT controls. It is possible that NMNAT2 may not be as protective against paclitaxel-induced neurite loss or axonal degeneration compared to vincristine based upon results of our *in vitro* studies. A greater magnitude of depletion of NMNAT2 may be required to impair cell viability in response to paclitaxel compared to vincristine treatment. The failure to detect an impact of a 50% reduction in NMNAT2 on paclitaxel-induced decreases in cell viability in cortical cultures derived from NMNAT2+/- mice may also reflect the fact that the MTT assay probes mitochondrial function as a measure of overall cell viability [18] see also [31]. It is unlikely that compensatory changes would occur as there were no differences in other NMNAT isoforms or associated proteins within the DRG. Further, no changes in NMNAT1 levels are observed following NMNAT2 reduction in brains of rTg4510 mice prior to neurodegeneration [32].

To ascertain whether floor effects could contribute to our failure to observe behavioral differences between NMNAT2+/- and WT mice in either the development or maintenance of chemotherapy-induced peripheral neuropathy, we challenged mice of both genotypes with an intradermal injection of capsaicin. Capsaicin, an agonist for the transient receptor potential cation channel subfamily V member 1 (TRPV1), produces an acute inflammatory pain state that manifests behaviorally as nocifensive behavior immediately following the injection, followed by the development of hypersensitivity to both heat and mechanical stimulation [33,34]. Capsaicin can also induce distal axonal degeneration, which may involve roles of NMNAT family proteins [35]. In our study, intradermal capsaicin challenge further enhanced mechanical allodynia in paclitaxel-treated mice, but the magnitude of capsaicin-induced hypersensitivity did not differ in paclitaxel-treated NMNAT2+/- and WT mice. Moreover, capsaicin-evoked responding was comparable in NMNAT2+/- and WT mice that were not subjected to chemotherapeutic treatment. Thus, 50% reduction in NMNAT2 levels did not alter responsiveness to a capsaicin-induced inflammatory challenge regardless of the presence or absence of chemotherapy pretreatment. Thus, maximal levels of paclitaxel-induced hypersensitivity did not prevent us from detecting detrimental effects of NMNAT2+/- reduction on paclitaxel-induced allodynia.

In conclusion, complete loss of NMNAT2 in cortical neurons renders them sensitive to neurotoxicity induced by vincristine, and to a lesser extent, paclitaxel treatment. Vincristine also produced concentration dependent losses in cell viability in NMNAT2+/- cortical neurons. By contrast, adult NMNAT2+/- mice did not exhibit detectable deficits in either the development or maintenance of chemotherapy-induced neuropathic pain produced by toxic challenge with either a vinca alkaloid (vincristine) or a taxane (paclitaxel)-based chemotherapeutic agent. Similar levels of chemotherapy-induced hyperresponsiveness to both mechanical and cold stimulation were observed in NMNAT2+/- and WT mice for each type of toxic neuropathy. The failure to detect changes in chemotherapy-induced peripheral neuropathy in adult NMNAT2+/- mice cannot be attributed to compensatory changes in other NMNAT isoforms, NMNAT enzyme activity or changes in MAP2 or neurofilament levels in adult DRG. Furthermore, no differences in responding were observed between NMNAT2+/- and WT mice when either neuropathic or non-neuropathic animals were challenged with capsaicin. Motor function in the rotarod test also did not differ between genotypes. Our results suggest that a 50% knockdown of the NMNAT2 protein does not impact chemotherapy-induced peripheral neuropathic allodynia as measured by responsiveness to mechanical and cold stimulation. Future studies employing a conditional knockout of NMNAT2 or associated proteins may be required to fully elucidate any impact of NMNAT2 on chemotherapy-induced peripheral neuropathy.
